# Supplementary material for: Who’s who in Magelona: phylogenetic hypotheses under Magelonidae Cunningham & Ramage, 1888 (Annelida: Polychaeta)
Source: PeerJ. 2021 Sep 21;9:e11993. doi: 10.7717/peerj.11993 (PMC8759375; doi:10.7717/peerj.11993)
Supplement: Supplemental Information 1 [file peerj-09-11993-s001.docx]

**Table S1.** **Character matrix of Magelonidae taxa and characters.** Outgroups are *Phyllochaetopterus limicolus*, *Spio filicornis* *Laonice cirrata*, *Prionospio lighti*, and *P*. *ehlersi*. ‘?’ = unknown; ‘-’ = inapplicable; ‘*’ = polymorphism, 0/1, in *Magelona* sp. H.

**1 2 3 4 5 6 7**

**0 0 0 0 0 0 0**

*Phyllochaetopterus limicolus* 01----20-0 0--00-0-0- ---------- 0-1------- ---------0 0000-----0 -----0---- --000-001

*Spio filicornis* 01----00-0 0--00-1-1- 0100-0-1-0 0-10100001 0010000100 000000-000 -----10-0- 11100-001

*Laonice cirrata* 01----20-0 0--00-1-1- 0100-0-1-0 0-00100001 0110001100 000000-001 0001012-0- 01100-00?

*Prionospio lighti* 01----00-0 0--00-1-1- 0100-0-1-1 0-10100001 0110001100 000000-000 -----12-0- 01100-00?

*P. ehlersi* 01----00-0 0--00-1-1- 0100-0-1-1 0-00100001 0110001100 000000-001 0001012-0- 01100-00?

*Magelona alexandrae* 1000110111 1101111111 0111110001 10?0111110 111002101? 0001110101 000111100- 10000-110

*M*. *alleni* 1002101111 1221111010 10-0-10001 10010-0100 110-011000 010000-010 -----1100- 000010111

*M. annulata* 1000111111 1??1111010 1100-1?001 10011001?0 1110011010 0000111000 -----101?? 100?0-??0

*M. anuheone* 1000110111 1111111110 0101110001 10?0101100 111001100? 0001111101 0????1110- 10010-1?0

*M. berkeleyi* 1000112111 1111111111 0101111001 1120101210 0110021010 0100110100 -----1110- 000?0-?00

*M. californica* 10020-1101 1??1111010 00-0-0-000 ??000-0000 010-001000 000000-000 -----1010- 10000-110

*M. cepiceps* 1002101111 1111111011 0101111001 ??00101110 0110111010 0100110100 -----1100- 00000-110

*M. cerae* 10001111?1 1??1111111 00-111?001 ?0200-1000 1100001000 000000-000 -----1000- 11000-110

*M. cincta* 1002101111 1111111111 10-0-11012 10?10-0000 110-001001 010000-001 101001100- 000010111

*M. cinthyae* 1000110111 1101111111 0101110001 10?0101210 111002101? 0001110101 00?1?1110- 10010-110

*M. conversa* 10010-0111 1221111111 010102-001 10?0101211 0010021011 1000010001 211001000- 01010-010

*M. cornuta* 1010111111 11?1111111 0101110001 10?0101210 111002101? 0000110101 000111100- 00010-110

*M. crenulata* 10010-01?1 11?1111111 012112-001 1000121210 0011000101 1000010101 2110?1100- 10000-?10

*M. crenulifrons* 1010111111 1??1111111 0101110001 1000101210 1110021010 0000111101 101001000- 00010-110

*M. dakini* 1002100111 1101111011 00-0-11001 1000100210 0110011010 0000111101 1012?1110- 00010-?10

*M. debeerei* 10010-0111 11?1111111 0101010001 ??00101211 0010000101 100000-001 ?110?1100- 01000-??0

*M. equilamellae* 1002102101 1111111011 10-0-11000 10010-0000 010-001000 010000-000 -----1100- 000010111

*M. falcifera* 10020-1101 1111111010 00-0-0-000 ??000-0000 010-00100? 0000010000 -----10110 10000-110

*M. fauchaldi* 10020-1101 1001111010 00-0-0-000 10000-0000 010-001000 000000-000 -----1200- 10000-110

*M. filiformis* 1002100111 1111111111 00-1110001 10000-1110 110-011010 0000111001 101201110- 00000-110

*M. gemmata* 1000110111 1101111111 0101110001 ???0101110 1110021010 0000110101 000101100- 10010-110

*M. hartmanae* 1002100111 1101111111 00-112-001 10000-1200 110-021010 2000111001 1011?1110- 10000-?00

*M. hobsonae* 1002102111 1111111111 00-1110000 10010-1210 110-011010 2000111000 -----1110- 00000-?00

*M. johnstoni* 10010-0111 1111111111 0111010001 1?01101210 0010000101 1000010001 211001100- 01010-010

*M. jonesi* 1022101111 12?1111011 0100-0-1-0 1010100110 0110001100 0100110000 -----1010- 01000-?00

*M. lenticulata* 1010112111 11?1111111 010111?0?1 10211010?1 0110121010 0100110000 -----1100- 000?0-1?0

*M. lusitanica* 1000110111 1101111111 0101110001 ??00101210 1110021010 0000110101 101001100- 10000-110

*M. magnahamata* 10020-1101 1001111010 00-0-0-000 ???00-0000 010-00100? 0000010100 -----10110 100?0-110

*M. mahensis* 1002100111 1201111011 00-0-10001 ???00-0110 010-00100? 000010-001 101101000- 00000-110

*M. marianae* 1000111111 11?1111111 011111?001 10101111?0 1110121010 000010-000 -----1000- 00010-?10

*M. minuta* 10020-1101 1001111010 00-0-0-000 0-000-0000 010-001000 0000010000 -----1000- 10000-110

*M*. *mirabilis* 10010-0111 1101111111 0100-2-1-1 1000100200 0010000101 1000010101 001201100- 01010-010

*M*. *montera* 1000110111 1101111111 0111110001 ???0111110 111002101? 0000111101 000111110- 00000-110

*M*. *nonatoi* 10021011?1 11?1111011 0101111001 1000101110 0110111010 0100110000 -----1110- 00000-110

*M*. *obockensis* 10010-0111 1111111111 0121111001 1001121210 0011021011 1000010101 211001100- 000?0-1?0

*M*. *pacifica* 1000110111 1111111111 0101110001 1110101110 1110021010 000011?101 000111000- 0?000-110

*M*. *papillicornis* 10020-1101 1??1111010 00-0-0-000 0-000-0000 010-001000 0000010000 -----1010- 10000-?10

*M*. *parochilis* 10010-0111 1111111111 0101111001 0-11101210 0010021011 100000-001 211001100- 01000-110

*M*. *paulolanai* 1000111111 1101111111 0101110000 10?01012?0 111002101? 000?110101 00?0?1100- 10000-110

*M*. *pettiboneae* 10020-1111 1101111010 00-0-0-000 0-000-0000 010-001000 0000110000 -----1010- 00000-110

*M*. *phyllisae* 1002111101 1101111010 00-0-0-000 0-000-0000 010-001000 0000111000 -----1010- 10000-?00

*M*. *pitelkai* 1002100111 1111111111 10-1111001 11010-1210 1110021010 2000111101 1011?1110- 00010-?0?

*M*. *polydentata* 10020-2101 1211111011 10-0-1?000 10200-0100 010-011000 011000-000 -----1200- 000?10111

*M*. *posterelongata* 1000110111 1??1111011 10-0-10001 10010-0100 110-011000 0001010010 -----1010- 00000-?10

*M*. *pulchella* 1000111111 1101111111 0101110001 ??01101210 1110021010 0000110101 202001000- 00000-110

*M*. *pygmaea* 10020-1101 1001111010 00-0-0-000 ??000-0000 010-00100? 000000-000 -----1100- 100?0-110

*M*. *riojai* 10010-0111 1111111111 1101111001 1101101211 0010000101 1000010101 101??1100- 01000-?10

*M*. *sacculata* 10010-0111 1?01111111 0100-1?1-1 1011100??1 0010000101 1000010011 2110?1100- 01000-??0

*M*. *sinbadi* 1000110111 1101111111 0101111001 ???0101210 0110021010 0100111100 -----1100- 00000-110

*M*. *spinifera* 1010111111 11?1111111 010111?1-1 ??00101210 0110021010 0?001110?0 -----10?11 1?010-?10

*M*. *symmetrica* 1022102101 1211111010 10-0-0-000 ???10-0000 010-00100? 010000-000 -----1100- 000011111

*M*. *tehuanensis* 1010111111 1??1111111 0101110001 1000101210 1110021010 0?00110000 -----1100- 10010-?10

*M*. *tinae* 10010-0111 1111111111 0111111001 ???0121210 001102101? 100?010101 211001100- 000?0-110

*M*. *uebelackerae* 1000110111 1??1111011 00-0-10001 10100-01?0 010-011010 0000110000 -----10110 10000-??0

*M*. *variollamelata* 1002102101 1221111011 10-0-11001 11210-0100 110-011000 0110010000 -----1100- 000010??1

*M*. *wilsoni* 1010112111 1101111011 0101111001 1020101210 0110121010 0100110000 -----1100- 000?0-110

*M. guineensis* 1002101111 ???1111111 10-1110001 ??010-1100 110-011010 0100010000 -----1100- 00001011?

*M*. *picta* 1002102111 ???1111111 0101110001 10?0101110 010-00100? 0100110100 -----1100- 00001001?

*M*. *nanseni* 1002102101 1211111111 0101110001 ???0101100 010-00100? 0100110000 -----1100- 00001111?

*M*. *fasciata* 1002101111 1211111011 10-0-11011 11?10-0000 110-01101? 0100010001 101001000- 00001011?

*M.* *mackiei* 1002102111 1211111111 00-1110001 ???00-1110 010-01101? 0100010000 -----1000- 000011111

*M.* sp. A 10010-0111 1??1111111 012112-001 1001121211 0012021011 1000010101 2110?1000- 00000-?10

*M.* sp. B 10010-0111 1??1111111 012112-001 1001121210 0011000101 1000010001 2110?1100- 00000-??0

*M.* sp. C 10020-1101 1??1111010 00-0-0-000 0-000-0000 010-001000 0000110000 -----10110 10000-??0

*M.* sp. D 1010111111 1??1111111 01011101-1 1000101210 1110021010 0?00111000 -----10?11 10010-??0

*M.* sp. E 1010110111 1??1111010 10-1110001 10000-1100 010-111010 0000110000 -----10?12 10000-?00

*M.* sp. F 1012101111 1??1111010 00-0-1?001 10000-0100 010-001000 0000010000 -----1000- 00000-?00

*M.* sp. G 1000110111 1??1111111 0101110001 1010101110 1110021010 0000110101 0000?1010- 00010-?10

*M.* sp. H 1000111111 1??1111110 0100-10001 10000-0100 1110011000 0?01111100 -----1*110 00000-?00

*M.* sp. I 1002102101 1??1111011 10-0-1?001 10210-01?0 110-011000 0110010000 -----1200- 000010???

*M.* sp. J 1012112111 1??1111011 0101111001 1020101210 0110121010 0100110000 -----1100- 00000-?10

*M*. sp. K 1000110111 1??1111111 0101111001 1000101110 1110021000 0?00111100 -----1100- 00000-?10

*M.* sp. L 1010111111 1??1111111 0101110001 1000101210 0110011010 0?00110101 0000?1100- 10010-?10

*Octomagelona bizkaiensis* 100210210? ???1101111 10-0-0-001 10210-0000 ---------- -10000-000 -----1100- 10000-100

*O*. sp. W Africa 1002102101 ???1101111 10-0-0-001 ??20?00??? ---------- -10?0????? ?????1??0- ??0?0-100
